# 2019-nCoV - Towards a 4th generation vaccine 

**DOI:** 10.6026/97320630016139

**Published:** 2020-02-12

**Authors:** Francesco Chiappelli

**Affiliations:** 1 UCLA Center for the Health Sciences, Los Angeles, CA, USA, Francesco

**Keywords:** Angiotensin-converting enzyme-2 (ACE2), transcription regulatory networks (TRNs) Corona virus (CoV), Middle-East Respiratory Syndrome (MERS), Severe Acute Respiratory Syndrome (SARS), Novel Coronavirus (emerged late) 2019 (2019-nCoV Corona Virus Disease 2019 (COVID-19)

## Abstract

The first report of the unusual manifestation of pneumonia-like symptoms in Wuhan City, China was made on 31 December 2019. Within one week, the Chinese authorities reported that they had identified the causative agent as a new member of the Coronavirus family, the same family of that was responsible for MERS and SARS not so many years ago. The new virus was called Novel Coronavirus 2019 (2019-nCoV). Three weeks later, the World Health Organization declared that 2019-nCoV was capable of direct human-to-human transmission, the virus had spread across several countries in three continents, and had infected close to two thousand people, of whom at least 1 in 5 quite severely. The number of fatalities was fast rising. Yet, the World Health Organization officially announced that there is still at present no recommended anti-nCoV vaccine for subject at-risk, nor treatment for patients with suspected or confirmed nCoV, let alone 2019-nCov. It is therefore timely and critical to propose new possible and practical approaches for preventive interventions for subjects at-risk, and for treatment of patients afflicted with 2019-nCov-induced disease (Corona Virus Disease 2019; COVID-19) before the present situation explodes into a worldwide pandemic. One such potential clinical protocol is proposed as a hypothesis.

## Background

On 31 December 2019, the World Health Organization was alerted to several cases of pneumonia in Wuhan City, Hubei Province of China. The causative pathogen was suspected to be a virus, but it did not match any other known virus. The following day, Wuhan City officials closed the Huanan seafood market, suspected to be the source of the mystery pathogen, because it was reported that certain patients presenting with the symptoms were vendors at that public market. By January 4 2020, the Chinese Health Organization reported 44 active cases. On 7 January 2020, Chinese authorities confirmed that they had identified the causative agent as a novel Coronavirus (CoV). That family includes viruses of the common cold as well as viruses known to cause Middle-East Respiratory Syndrome (MERS); Severe Acute Respiratory Syndrome (SARS).The new CoV was named Novel Coronavirus (emerged late) 2019 (2019-nCoV). Two days later, Chinese authorities reported the first fatality linked to 2019-nCoV: a 61-year-old male who had been admitted in the first cohort of patients. He had several other underlying medical conditions, which may have contributed to weakening his immune system. Apart from respiratory failure and severe pneumonia caused by 2019-nCoV, the patient suffered from abdominal tumors and chronic liver disease. On 12 January, Chinese scientists released the genetic sequence of 2019-nCoV, in part because nonofficial report of international spread of 2019-nCoV had commenced. The next day, Thailand officially reported its first imported case of 2019-nCoV: a 61-year-old woman from Wuhan – she, however, denied having visited the Huanan seafood market. On January 15 2020, Chinese authorities reported the second death attributed to 2019-nCoV: a 69-year-old male who also suffered of other unrelated severe pathologies, including myocarditis. Infection with 2019-nCov, nonetheless, were thought to be responsible for his abnormal renal function, and severely damaged to multiple organ functions. The following day, Japan reported its first case of 2019-nCoV: a Chinese man in his 30s, who also denied having visited the Huanan market. On January 17, Thailand confirmed the second imported case of 2019-nCoV. Chinese authorities noted a spike in 2019-nCoV infections between January 18 and 19, 2020. That observation arose the suspicion that 2019-nCoV was capable of direct human-to-human transmission. The following day, 20 January 2020, South Korea confirmed its first case of 2019-nCoV infection: a male patient who denied having visited any public markets in Wuhan. On January 21 2020, the World Health Organization confirmed human-to-human transmission of 2019-nCov. As of that date, the total official number of cases has risen to 222, although it was suspected to be in reality much higher. Infection had spread to health-care workers, and it was suspected that one mode of transmission may be via the eye mucosa. Chinese authorities have also reported a fourth death. The situation was fast becoming alarming: suspected cases appeared in France, Italy and other countries of Europe.Australia seems to be affected as well.Other countries in Asia also reported suspected cases, including the Philippines and Singapore. Suspected cases of 2019-nCoV were reported in North America. The following day, 22 January 2020, World Health Organization Director-General Tedros Adhanom Ghebreyesus convened an emergency meeting to decide whether 2019-nCoV should be declared to constitute a worldwide public health emergency of international concern.Despite a significant rise in confirmed cases of individuals infected with 2019-nCoV – in China alone, at 580 infected individuals, with a death toll now at 17 in the Hubei Province alone - the emergency committee deferred its decision on whether to advise Director-General Ghebreyesus to declare the 2019-nCoV outbreak a public health emergency pandemic of international concern. On January 23, Chinese authorities shut down the city of Wuhan: no public transportation, closed airport and railway station for 11 million people. Later that same day, the city of Ezhou is also in complete lockdown. Festivities for the upcoming Chinese New Year were cancelled throughout China to minimize human contact in crowds.

The following day, the city of Huanggang was declared under lockdown. Singapore confirmed its first imported case, and Vietnam confirmed two cases. Director-General Ghebreyesus declared that, indeed, the 2019-nCoV outbreaks is a public health emergency of international concern. On January 24 2020, the official number of confirmed cases of patients infected with 2019-nCoV had risen to 830 in China alone, with 177 (21%) among them in severe and critical condition. The number of fatalities caused by 2019-nCoV in China was now 25. Japan confirmed its second 2019-nCoV case. Nepal confirmed its first case. The following day, Australia confirmed its first case of 2019-nCoV, as did France. Two suspected cases in Italy were being closely monitored. In China, the official number of new infections – that is, over the previous 24 h – was 444, and the number of new deaths was 16 above and beyond the number reported the previous day. The official number of individuals confirmed to be infected with 2019-nCoV in China became 1,287, including 237 (20.7%) in severe and critical condition.

Fears of an exponential rate of growth of 2019-nCoV has begun to mount among public health specialists worldwide as the total of deaths from 2019-nCov in China has risen to 41. Infection with 2019-nCoV is now fast spreading across continents. At least two new cases have been reported and confirmed in the United States. And it has just been one month yet since the first alert. The causative agent for this alarming unfolding of events, 2019-nCoV, is a corona virus related to the viruses that have caused important outbreaks in the past, including the Middle-East Respiratory Syndrome (MERS), and the Severe Acute Respiratory Syndrome (SARS) [1].There is no first-second-or third-generation vaccine available for any members of the Cov family, nor is there practically the time to develop, raise, test and evaluate the effectiveness of a vaccine for 2019-nCov. Moreover, the World Health Organization stated in its 12 January 2020 recommendations entitled ‘Clinical management of severe acute respiratory infection when novel coronavirus (nCoV) infection is suspected - Interim guidance; WHO/nCoV/Clinical/2020.1' that "...there is no current evidence from RCTs to recommend any specific anti-nCoV treatment for patients with suspected or confirmed nCoV…". In brief, the international medical community is totally devoid of tools to combat the unfolding 2019-nCov thereat to global public health – not in terms of preventive medicine to protect subjects at-risk, and not in terms of clinical interventions for infected patients.

What is known, however, is that 2019-nCov, like all corona viruses belong to the Coronaviruses (Coronaviridae) family of RNA viruses that cause diseases in mammals and birds that include diarrhea in cows and pigs, and upper respiratory disease in chickens. In humans, the virus causes respiratory infections, which are generally often mild, rarely lethal. The trends we begin to observe with 2019-nCov suggest that it can be directly transmitted human-to-human, and that it causes serious infections in roughly one in five patients that can lead to death: staggering preliminary statistics. Previous research with other CoV members indicates that proteins of Coronaviruses that could be used in the generation of vaccines include the spike, the envelope, the membrane and the nucleocapsid proteins. The spike protein is of particular interest because it is responsible for the penetration of the virus into the cell, which leads to the initiation of viral replication and proliferation. The spike protein binds to the angiotensin-converting enzyme 2 (ACE2) transmembrane - receptor on the eukaryotic host cell. Case in point, SARS-CoV binds to ACE2, as does MERS-CoV [2]. Indeed, ACE2 is the obligate cellular receptor for CoV entry process via the spike protein [3].

While the development of a vaccine of the 1st, 2nd or 3rd generation against the spike protein is possible but time consuming, it is therefore timely ad critical to propose new possible and practical approaches for preventing infection of subjects at-risk and for treatment intervention of patients infected with 2019-nCov,or any other CoV for that matter.One such alternative protocol is proposed below.

## Methodology:

Short of 1st, 2nd or 3rd generation vaccine measures for preventive CoV, and short of clinical treatment interventions for patients infected with CoV, and specifically, 2019-nCov, it is timely and critical to evaluate new alternatives. Here, we propose that one putative 4th generation vaccine to control 2019-nCoV explosion might simply involve the genetic engineering a soluble binary molecule (i.e., ACE2R-ACE2R; [ACE2R] 2) or its quaternary form (i.e. two intertwined ACE2R-ACE2R; [ACE2R] 4). This process is fast, reliable and precise by today's standard, and doable in any modern biochemistry laboratory. The obtained sterile molecule could be injected in individuals at high risk as a preventive 4th vaccination measure, or as a treatment intervention in confirmed cases of 2019-nCoV infection. The soluble molecule is expected to bind the spike protein of circulating CoV with higher affinity than the transmembrane ACE2R, and to render the CoV particles, therefore, incapable of binding to the cell receptor, of penetration into the cells, and of replicating inside the cell. The proposed 4th generation vaccine would, besides protecting the cells from CoV infection, also preserve ACE2 intracellular functional activity, and guard against the rise of serum angiotensin II levels, which can be pathogenic to lung cell integrity. In brief, the 4th generation vaccine proposed here would prevent at-risk individuals from becoming sick from any incipient infection: that is, in the true meaning of the term, it would 'vaccinate' them against CoV in general, and in the present case of high emergency provide substantial protection against2019-nCoV. Moreover, should the molecule be genetically engineered to incorporate a neutral protein, such as human serum albumin, the soluble albumin-[ACE2R] 2 or albumin-[ACE2R] 4 complex injected in 2019-nCoV-infected patients would bind the circulating CoV. Patients could then undergo a treatment intervention of 'cleaning' their blood from albumin- [ACE2R] n-CoV complexes by a clinical protocol akin to dialysis. The patient's blood would be passed through a sterile column constructed with high affinity anti-human albumin antibodies. The anti-albumin antibody-albumin- [ACE2R] n-CoV moieties would be retained on the column, and the 'CoV-cleaned’ blood returned to the patient to dampen the infection. It is possible that the binding of CoV spike protein to ACE2 is a down regulation of its expression, resulting in increased serum angiotensin II levels, and lung injury. Indeed, administration of recombinant human ACE2 in experimental models of CoV infection ameliorates lung injury in animal models [4]. Therefore, we propose that the 'CoV-cleaned' blood returned to the patient would also be enriched with recombinant human ACE2 to ameliorate lung injury.

## Discussion:

Vaccines that are raised from whole pathogens - attenuated or inactivated – are called 1st generation vaccines.Protocols that involve utilizing specific protein components extracted from the pathogens to reduce risks and side - effects in the host produce 2nd generation vaccines. By contrast 3rd generation vaccines are vaccines derived from administration of genetically engineered DNA or mRNA to induce the host cells to produce an antigen in vivo, which in turn is expected to be recognized as non-self, and generate protective antibodies [5]. Here, we propose a new avenue in vaccinology: the generation of a molecule with the purpose of preventing infectious disease - that is, a vaccine -, but not based on the traditional norms of antigen-idiotype binding. The 4th generation vaccine we theorize here depends upon the specificity of receptor-ligand binding, but is a biochemical molecule constructed artificially for the purpose and intent of neutralizing the infectious agent.CoV in general, and 2019-nCov may not escape immune surveillance as other viruses often do [6- 8]. Nonetheless, it follows that raising a 1st or 2nd generation vaccine against the CoV spike protein could be considered a viable protocol in the development of preventive measures to counter CoV-induced epidemics and pandemics. But the timeline to generate and test such vaccine species in the present emergency of the 2019-nCoV pandemic is be prohibitive, as it would also be should we witness a contemporary resurgence of the SARS or MERS epidemic. Moreover, the utility of live-attenuated vaccines CoV is limited by risks of reversion or repair [8]. Alternative 3rd generation CoV vaccines may be theoretically promising in that CoV with completely rewired transcription regulatory networks (TRNs) are effective vaccines against SARS-CoV, and putatively MERS-CoV. TRN-rewired CoV are attenuated and protect against lethal SARS-CoV challenge [9].TRN-rewired CoV are neither, properly speaking, 1st or 2nd generation vaccine, and neither are they 3rd generation vaccines:they are efficacious hybrid measures that prevent or slow down SARS-CoV, and possibly MERS-CoV epidemic. However, the urgency of the present moment precludes the somewhat lengthy experimentation time that would be required for the development and testing of a 3rd generation vaccine of the sort. Since scientists have had several issues up to this point in the process of producing a 3rd generation vaccine for SARS or MERS, whose epidemics were several years ago, it implausible that they could now develop such a 3rd generation vaccine for 2019-nCov in the emergency the world is experiencing today.

## Conclusions:

**Figure 1:**
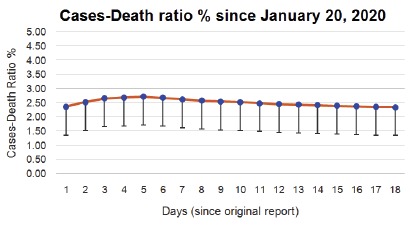
2019-nCoV Infection, China (data from BNO News, The Netherlands; bnonews.com) Confirmed Infection Cases-Fatalities Percent in China

Taken together, the important points brought forth above emphasize the fact that the field of vaccinology cannot and must not be limited strictly to 1st, 2nd or 3rd generation vaccines. A 4th generation of vaccines is now emerging that may seem unconventional, but converge toward the same goal of preventing the spread of infectious disease. These 4th generation vaccines may be particularly relevant in the case of flaming epidemics, when the time to generate, test, evaluate and distribute 1st, 2nd or 3rd generation vaccines is prohibitive, such as is precisely the case now with 2019-nCoV. In certain circumstances, public health urgency demands immediate intervention, and precludes the time required to generate and test new vaccine species.Case in point, the threat now posed by the new member of the Coronavirus family (2019-nConV), whose discovery was announced by the Chinese health authorities on Chinese authorities reported having isolated a new type of coronavirus on 7 January 2020. Whereas 2019-nCoV is reported to a beta coronavirus closely related to SARS and other coronaviruses that originate from bats, it is unclear – and at this point almost irrelevant - to date if 2019-nConV originated from bats or from snake or other animals and subsequently transferred to bats. What is clear is that 2019-nConV is capable of direct human-to-human transmission, and its infection patterns grows alarmingly fast across all continents. To be clear, three weeks into its original reporting, 2019-nCoV has infected children, men, women and elderly in all continents. In China alone, the number of confirmed cases are over thirty-seven thousand infected individuals (n=37,593 as of day 21), and the number of fatalities from the disease has risen over eight hundred (n=813).Whereas both the percent confirmed cases and the percent death rate seem to have steadily decreased in parallel over the past 21 days, the case-fatality percent rate has remained steady above 2% (mean ± SD: 2.34% ± 0.39) (Figure 1). As a reference point, the case-fatality percent rate of the Spanish influenza following World War I worldwide was at, or slightly above 2.5%; that same statistic for measles with no preventive vaccination measures is close 15%.

In brief, 2019-nCoV seems to be less lethal than the Spanish flu, and may be abating somewhat at its original epicenter; it has generated heightened fear for a global pandemic as other epicenters have emerged, including Singapore and Thailand. In this hypothesis report, we have proposed here a new avenue into 4th generation vaccines. Thus, vaccine protocols that do not involve the generation of antibodies against whole pathogens uses protein extracts obtained from pathogens, or nucleic acids related to pathogens. Rather, the preventive and protecting ability of the intervention we propose, which still relies on the specific binding of the pathogen to a substrate generated specifically against it, is a biochemical construct, which could actually best be generated by artificial intelligence of immune surveillance [8] algorithms in the not so distant future. The construct we propose here, specific to CoV, and applicable to 2019-nCoV in the context of the immediate urgency that is upon us, can be generated and expanded quickly, simply and reliably in any biochemistry laboratory. We also describe how it can be effectively utilized in treatment protocols of patients already infected with 2019-nCoV, in a slight modification of the common clinical protocol for renal dialysis.

## References

[1]  Cui J (2019). Nat Rev Microbiol..

[2]  Li F (2013). Antiviral Res..

[3]  Kuhn JH (2007). Antivir Ther..

[4]  Zou Z (2014). Nat Commun..

[5]  Chapman R, Rybicki EP (2019). Vaccines.

[6]  Chiappelli F (2014). Bioinformation..

[7]  Chiappelli F (2015). J Transl Med..

[8]  Chiappelli F (2018). Bioinformation..

[9]  Graham RL (2018). Commun Biol..

